# How Joannites’ economy eradicated primeval forest and created anthroecosystems in medieval Central Europe

**DOI:** 10.1038/s41598-020-75692-4

**Published:** 2020-11-19

**Authors:** Mariusz Lamentowicz, Katarzyna Marcisz, Piotr Guzowski, Mariusz Gałka, Andrei-Cosmin Diaconu, Piotr Kołaczek

**Affiliations:** 1 Climate Change Ecology Research Unit, Faculty of Geographical and Geological Sciences, Adam Mickiewicz University, Poznań, Poland; 2 Faculty of History and International Relations, University of Bialystok, 15-420, Bialystok, Poland; 3 Department of Geobotany and Plant Ecology, Faculty of Biology and Environmental Protection, University of Lodz, 12/16 Banacha Str. 90-237, Lodz, Poland; 4 Department of Geology, Babes-Bolyai University, 400084 Cluj-Napoca, Romania

**Keywords:** Palaeoecology, Wetlands ecology

## Abstract

During European states’ development, various past societies utilized natural resources, but their impact was not uniformly spatially and temporally distributed. Considerable changes resulted in landscape fragmentation, especially during the Middle Ages. Changes in state advances that affected the local economy significantly drove trajectories of ecosystems’ development. The legacy of major changes from pristine forest to farming is visible in natural archives as novel ecosystems. Here, we present a high-resolution densely dated multi-proxy study covering the last 1500 years from a peatland located in CE Europe. The economic activity of medieval societies was highly modified by new rulers—the Joannites (the Order of St. John of Jerusalem, Knights Hospitaller). We studied the record of these directorial changes noted in the peat profile. Our research revealed a rapid critical land-use transition in the late Middle Ages and its consequences on the peatland ecosystem. The shift from the virgin forest with regular local fires to agriculture correlates well with the raising of local economy and deforestations. Along with the emerging openness, the wetland switched from alkaline wet fen state to acidic, drier *Sphagnum*-dominated peatland. Our data show how closely the ecological state of wetlands relates to forest microclimate. We identified a significant impact of the Joannites who used the novel farming organization. Our results revealed the surprisingly fast rate of how feudal economy eliminated pristine nature from the studied area and created novel anthroecosystems.

## Introduction

Ecosystems constantly change over time while their stability is just an impression in the short time window^1^. Ongoing global change are affecting biodiversity and functioning of nature^2^, while large-scale deforestation is one of the worst consequences of the growing economy in the Anthropocene^3^. However, the vanishing forest is not a new phenomenon. Humans started transforming nature in Europe and temperate regions 1000s of years ago^4,5^, increasingly changing landscape through deforestation and agricultural development^6,7^. Neolithic revolution and later intensified agriculture in the Medieval Age triggered cascading effects that led to critical transitions of the pristine ecosystems through the transformation of vegetation and soils^4,8^. These forest-agriculture transitions affected structures and functions of ecosystems^9,10^. Such patterns are especially apparent, for example, in data from northern and southern America where the arrival of Europeans significantly modified ecosystems and local populations in few centuries^11^. Consequently, pristine biomes of the planet were gradually transformed into anthropogenic biomes^12^. It is estimated that virgin nature was largely transformed by hunter-gatherers, farmers, and pastoralists by 3000 years ago^5^. In CE Europe, the first human impact signals recorded in paleoecological archives are dated up to 6000 years ago^13,14^.

Environmental history has been reconstructed from sedimentary archives using the multi-proxy approach^15,16^. An increasing number of paleoecological multi-proxy reconstructions revealed different types of disturbances of various ecosystems including wetlands^17,18^. In the Medieval Age, accelerating development of economy led to considerable land-use change that affected forests and wetlands. During the development of CE European states, various cultures utilized natural resources, but their impact was not equally spatially and temporally distributed. An intriguing example comes from Poland where considerable changes resulted in landscape fragmentation, especially during the Middle Ages, for example by strongly developing economy^19,20^, and the Teutonic Order^21–23^. However, there is not much evidence on how the arising economy and loss of pristine forest affected wetland ecosystems in a long temporal scale. The potential of wetlands to record the land-use change on the turn of tribe-to-state transitions has not been adequately addressed. Case studies of the forest–wetland interface have been scarcely conducted in appropriately high resolution. Among other ecosystems, humans tend to perceive wetlands as remnants of pristine landscapes that were not modified by rising anthropogenic pressure. In reality, wetlands are sensitive to even subtle transformation introduced in their catchment^24,25^ as well as to direct modifications by humans, e.g., exploitation or drainage^26^. Despite not being exploited by past societies that existed before the Middle Ages, wetlands (including peatlands) were indirectly impacted by various economic activities CE Europe, e.g., deforestation^24,27^ and eventually often degraded along with increasing human pressure^28^. In the last 300 years, European peatlands experienced hydrological stress related to climate and land-use change^28,29^. Nonetheless, several sites remained in a good state and avoided dry shifts in the recent centuries^30^.

Resilient forests and peatlands possess at least fragment of the original biodiversity that has not changed over the last centuries^31–33^. Ecological assessment of baseline conditions of individual sites is possible only through paleoecological reconstructions of long-term environmental changes, and surface vegetation surveys are usually not sufficient^18,34,35^. Wet and fast-growing peatlands surrounded by disturbed forests are very rare, but they are an excellent archive of the history and changes in nature. The thorough multi-proxy high-resolution peat records have rarely been presented in the light of the historical turnovers. We fill this gap by exploring the 1500-year history of the land-use change and the well-known settlement history supported by the rich historical survey. The studied site is located in close vicinity of Łagów—a village founded in the early thirteenth century that was inhabited by different past societies preceding the Polish State and later settled by the Joannites (Order of St. John of Jerusalem, Knights Hospitaller) and transformed into the main town of the region. Considering the proximity of the town and the studied wetland, we hypothesized that economic transformation and related environmental changes associated with the tribe-to-state transition and medieval economy caused irreversible modifications in the forest structure with the cascading consequences for peatland functioning. By using the multi-proxy record, we aimed to (1) infer the effect of deforestations and fires on peatland development by focusing on the impact of the economic activity of the Joannites that stationed in the nearby Łagów town and exploited surrounding villages and (2) to reconstruct hydrological dynamics and its relation to human-induced vegetation changes.

### Site description

Pawski Ług is an ombrotrophic *Sphagnum*-dominated peatland located in western Poland in the Łagów Lakeland (52°19′45″N, 15°16′30″E). The area is covered by morainic hills and a number of small glacial lakes. The peatland covers an area of 3.67 ha, and its vegetation is currently dominated by *Sphagnum fallax, S. angustifolium*, and other species typical for raised bogs: *Drosera rotundifolia, Oxycoccus palustris, Eriophorum vaginatum*, and *Ledum palustre*. The peatland is surrounded by the mixed forest mainly composed of *Quercus*, *Fagus sylvatica*, and *Pinus sylvestris*. Pawski Ług is protected as a nature reserve since 1970.

## Results

### Regional historical background

**Figure 1:**
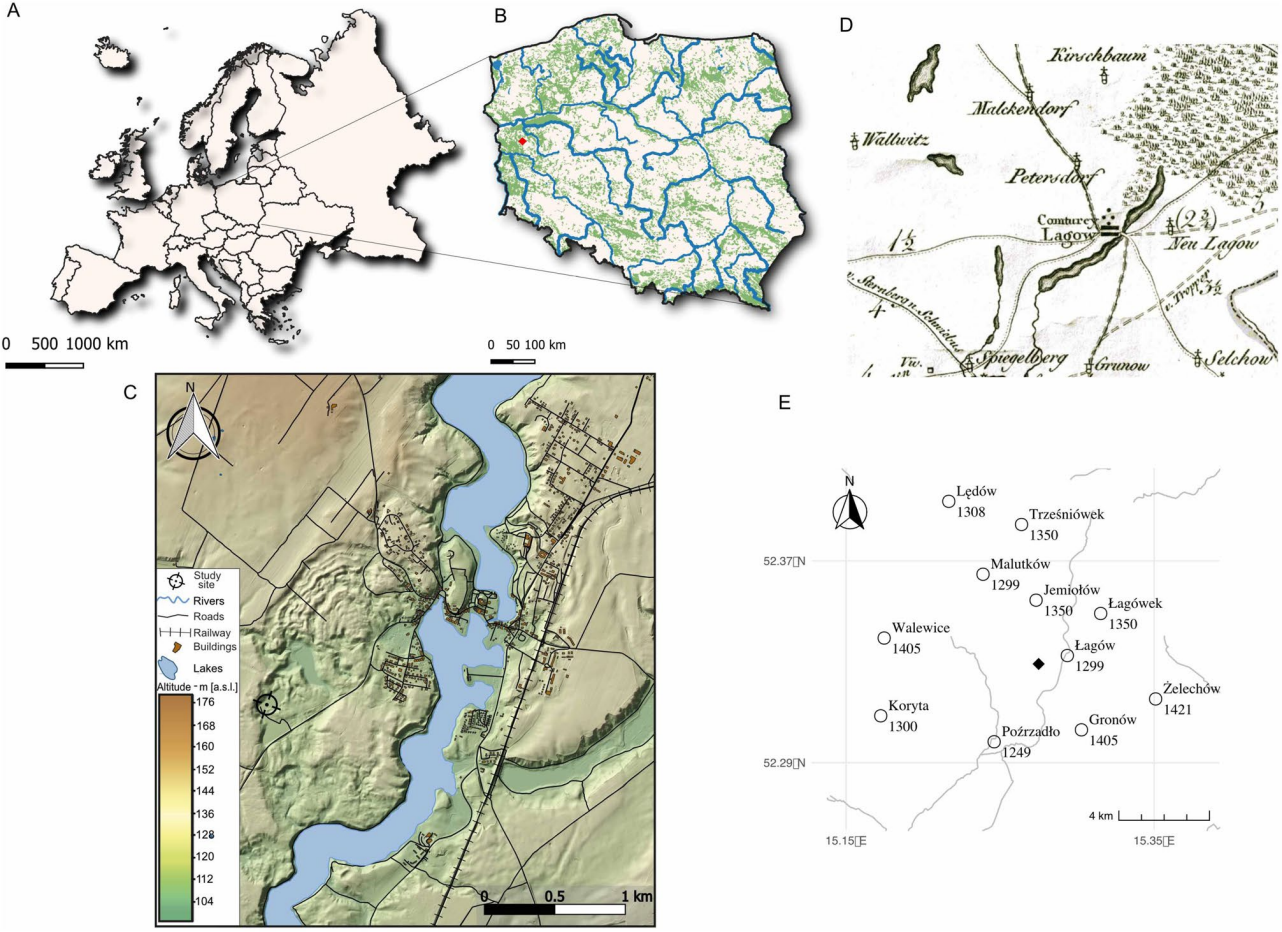
Location of the study site: (**A**) Europe, (**B**) Poland, the Pawski Ług peatland location is signed in red, (**C**) Coring site and surrounding areas, (**D**) Part of AI sheet, David von Gilly, Special Karte von Südpreussen: mit Allergrösster Erlaubniss aus der Königlichen grossen topographischen Vermessungs-Karte, 1:15,000 scale, ed. 1802–1803 (www.mapywig.org), (**E**) Settlements in the vicinity of the site with the date of their first appearance in written sources. Maps constructed by ML with Affinity Designer (https://affinity.serif.com).

Historical settlement structures near the Pawski Ług mire (Fig. 1) developed in the late Middle Ages as elements of a feudal estate with the central administrative unit in Łagów. The oldest traces of permanent settlement in the vicinity of the site (within a 5-km radius) relate to Łagów and the people of the Lusatian culture, which developed in this area between approximately 1300 BCE and 500 cal. BCE^36^. The first traces of Slavic settlement were found between 800 and 950 CE. It may have been the settlement of the Leubuzzi tribe, recorded in the work of the German chronicler Adam of Bremen. At the beginning of the second half of the tenth century, these areas were probably temporarily incorporated into the emerging Polish State, which was associated with the destruction of the network of older settlements of the Middle Oder region (hence perhaps noticeable traces of fire) and the construction of a new type of fortified settlements^37^. In 1124–1125 CE, Polish prince Bolesław Krzywousty founded a bishopric with the capital in Lubusz (German: Lebus) for the areas lying in the fork of the rivers Warta and Oder, which gave the name to the entire *Terra Lubucensis *region^38^.

In 1249–1252 CE, the area was granted in exchange for military aid by the Silesian Prince Bolesław Rogatka to the Archbishop of Magdeburg and the Magdeburg Margraves^39^. The latter built wooden fortifications (*castrum Lagowe*) along with a stone edifice (*Hus Lagow*) in Łagów for guarding the eastern border of Brandenburg^40^, and they started the process of colonization. It entailed the foundation of new villages or bringing new German settlers to villages previously inhabited by the Slavic population. By the mid-fifteenth century, the number of *Terra Lubucensis *settlements recorded in written sources increased from 61 to 175^41^. In 1347 CE, the Brandenburg Margrave Ludwig V pledged Łagów together with the adjacent property to the Knights of the Order of St. John and failed to repay the loan. In 1350 CE, he conferred on the Order the ownership of the manor house and town in Łagów and 21 other settlements. The Joanittes were an international order. Encouraging European elites to make pilgrimages to Jerusalem and support Christianity in Palestine, they also founded their houses in Central Europe. They also took possession of their estates in Brandenburg, Pomerania, and the Kingdom of Poland^42^. The Brandenburg Joannites established a commandery in Łagów, i.e., the headquarters of the basic unit of the organizational structure of the Order and built a castle there. In the late Middle Ages, there were 11 villages within a 5-km radius of the site, which became the Order’s property at the beginning of the fifteenth century^42^. All these formed a relatively stable settlement network in the following centuries. Joannites’ estate near Łagów was organised in a typical central European way. The Joannites performed the role of a feudal landlord, to whom Brandenburgian margraves granted land, and whose role was to modernize it in order to maximise the profits from agriculture, in the same manner as it was done in the estates of Brandenburgian, Pomeranian, Prussian and Polish members of nobility^43,44^.

The Joannites enlarged the brick castle and the bailey with houses of servants and craftsmen, thus creating a kind of district, next to which the old settlement on the slopes of the Falcon Hill functioned. Nearby villages were a part of the “table estate” of the Order, which means that in addition to providing the income in terms of money, they were supposed to supply the brothers with food. The lifestyle of the Joannites was more of a knight’s fraternity than of a church order, and in 1538 AD, they converted to Protestantism in Brandenburg (and in Łagów). They became a secular order and kept their property as an organization^42^. After the cessation of the Order of St. John in 1812, their property was transferred to the Prussian state, and from 1819, it was transferred to various aristocratic families, which at first continued to develop agricultural production in the area and the large manorial farms^45^, but environmental sources also indicate some changes in the trait of human economic activity.

### Multi-proxy, high-resolution peat archive: changes in forest and peatland ecosystems

**Figure 2:**
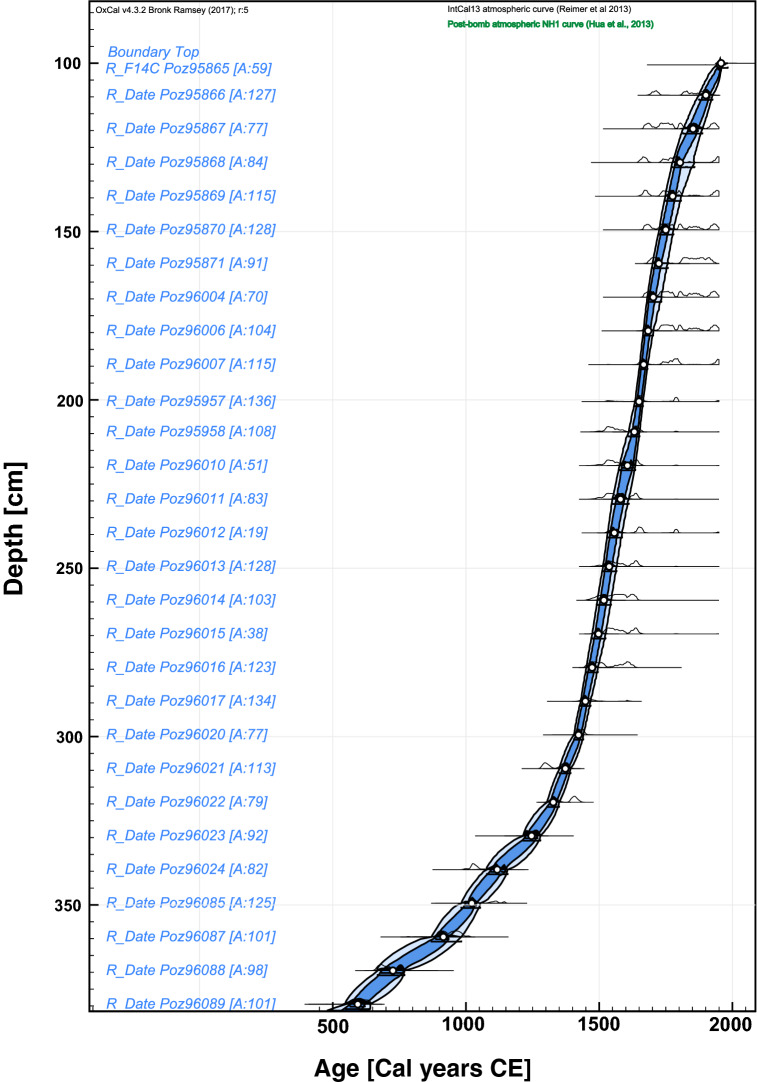
Age-depth model based on 29 14C dates, constructed in OxCal (https://c14.arch.ox.ac.uk). Figure constructed by ML with Affinity Designer (https://affinity.serif.com).

**Figure 3:**
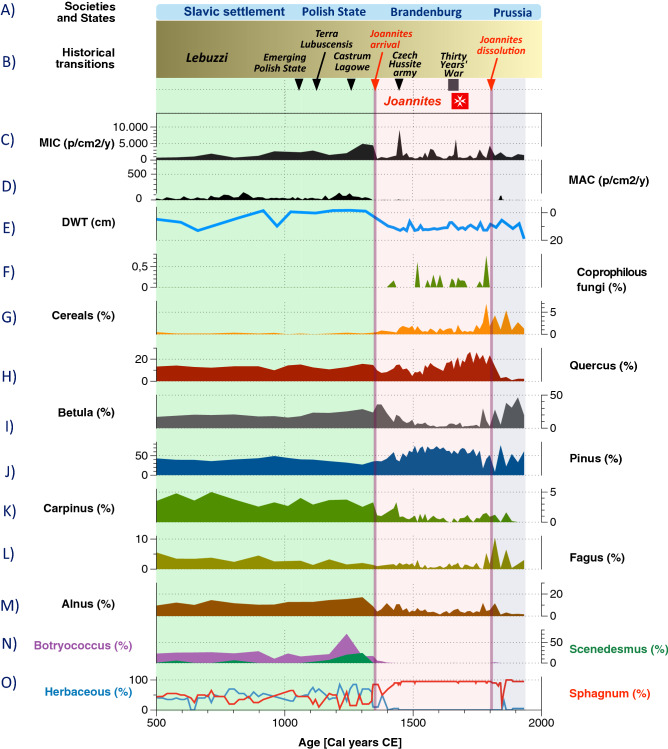
Synthetic diagram of the Głęboczek chosen proxies for the last 1500 years: (**A**) Past societies and states described in section Historical transitions: from tribe to state—SMOM (Sovereign Military Order of Malta), (**B**) Historical transitions in the analyzed region described in section historical transitions: from tribe to state—SMOM (Sovereign Military Order of Malta), (**C**) MIC—influx of macrocharcoal (particles/cm^2^/year), (**D**) MAC—influx of microcharcoal (particles/cm^2^/year), (**E**) DWT (cm)—testate amoebae-based ground water table reconstruction, (**F**) Coprophilous fungi—nonpollen palynomorphs (%), (**G**) Cereal pollen (%), (**H**) Quercus pollen (%), I) *Betula* sp. pollen (%), (**J**) *Pinus sylvestris* pollen (%), (**K**) *Carpinus betulus* pollen (%), (**L**) *Fagus sylvatica* pollen (%), (**M**) *Alnus* pollen (%), (**N**) Microalgae—*Botryococcus* and *Scenedesmus*—nonpollen palynomorphs (%), (**O**) plant macrofossils: herbaceous plants (%), *Sphagnum* (%). Figures constructed by ML DataGraph (https://www.visualdatatools.com/DataGraph) and Affinity Designer (https://affinity.serif.com).

By using the high-quality age-depth model (Fig. 2) and several proxies, we reconstructed three main stages of land-use change linked to peatland ecosystem functioning and structure. The synthetic Fig. 3 shows the synopsis of changes in the last 1500 years (Fig. 3). Individual proxy diagrams for pollen, testate amoebae (TA), and plant macrofossils are available as Supplementary Figures S1, S2, and S3, respectively. From ca. 500 to 1350 CE, herbaceous plants occurred together with *Sphagnum. *Moreover, *Nymphaea alba* sclereids were present in the entire phase, indicating open water habitats (Suppl. Fig. S2). TA communities changed abruptly (see Suppl. Fig. S3). The population of *Assulina muscorum* and *Amphitrema wrightianum* decreased. Other mixotrophs such as *Archerella flavum*, *Hyalosphenia papilio,* and *Heleopera sphagni* increased, and new species appeared in the communities, especially those typical for very wet habitats, for example, *Difflugia globulosa* and *Centropyxis aculeata*^46^. The water table, decreased slightly at ca. 500 cal. CE to ca. 15 cm and increased again reaching the surface of the mire. The reconstructed water table was the highest in the entire profile at ca. 900–1200 CE. Local vegetation was dominated by a dense forest with *Pinus sylvestris, Betula, Alnus,* and *Quercus*. *Corylus avellana* retreated in this phase, whereas *Fagus sylvatica* and *Carpinus betulus* expanded. A slight landscape opening is marked by the increase in Poaceae percentages, but arboreal pollen (AP) values were still very high (> 90%). The water table increase recorded in other proxy data is confirmed in pollen record by high numbers of Nymphaeaceae idioblasts and the sharp increase in *Botryococcus*^47^. The presence of coprophilous fungi indicates increased presence of herbivores/omnivores near the peatland^48^. Because of low frequency/lack of human pollen indicators, we assume that this telmatic stage was an effect of climate influence (Fig. 3). An increase in fire activity at the end of the phase coincides with water table lowering and a slight decrease in AP values (Suppl. Fig. S1). This gradual increase in charcoal and a decrease in forest vegetation can be the first stronger human signal in this record associated with the building of the Polish State.

In the stage of the peatland development from 1350 CE up to the present time, local vegetation was dominated by *Sphagnum*, mainly *S. fallax/angustifolium* and *S. magellanicum,* and *S. fuscum *in the final stage of this phase (Suppl. Fig. S2). Macro remains of species typical for open water habitats that were abundant in the previous phase, e.g., *Nympahea* *alba* sclereids, were not present. High peat accumulation rates in this phase and rapid peat growth (220 cm of peat accumulated over ca. 55 years) were probably related to the formation of a floating mat on the surface of the water basin, and *Sphagnum* growth was driven by autogenic succession. TA record estimates water table lowering as the reconstructed depth-to-water table (DWT) decreased to ca. 10 cm (Fig. 3, Suppl. Fig. S3). TA communities were highly dominated by mixotrophic species: *A. flavum*, *H. papilio*, *H. sphagni,* and *Hyalosphenia elegans*. Lower water tables are indicated by the presence of *Alabasta militaris*, a taxon present in dry conditions^46^, and *Arcella discoides* that prefers water table fluctuations and is often found in hydrologically unstable habitats^46^. Composition of the forests surrounding the site changed. The spread of *Pinus* *sylvestris* was identified at ca. 1350 cal. CE, and a retreat of *Betula* was noted, while *Quercus* became abundant. Moreover, AP values decreased toward the top of the profile. Among open land indicators, we noted an increase in Poaceae, *Plantago lanceolata,* and *Rumex acetosa *type., and the taxa typical for cultivated fields (*Secale cereale*, Cerealia type, and *Fagopyrum esculentum*) became more abundant. This was associated with late medieval colonization (Suppl. Fig. S3). However, the values of pollen human indicators do not suggest intensive pressure on the peatland. The decrease in *C. betulus* and *Alnus* and the increase in fire activity (expressed as an increase in microscopic charcoal influx and more dynamic charcoal curve) suggest that forest was exploited for timber and the wood was probably burned for household maintenance^49^.

After 1800 CE, forest regenerated as indicated by the pollen of *F. sylvatica* (presently surrounding the mire) and *Betula*. Moreover, the abundance of cereal pollen increased, which with the lack of coprophilous fungi suggest a change in land management from grazing to croplands.

### Environment and history: forest, farming, and critical transitions

#### Pre-Joannites: 500–1350 CE

Recorded historical phases reconstructed from the peat are well synchronized with the forest structure and fire intensity. The area experienced abrupt changes in land management. Pollen grains of the cereals appear in low percentage between 500 and 1350 CE. All local proxies revealed wet conditions in the mire and open water, suggesting the existence of the poor fen habitat. The characteristic feature of the Slavic societies occupying the region was a balanced relation with the forest. Archaeological examination of the entire region shows a concentration of inhabitancy in fortified settlements, around which most traces of open settlement were found^50^. The population density around our site was not high in the early phase. We inferred no sign of extensive deforestation. The mire was surrounded by the forest that locally burned, as shown by the macrocharcoal remains. A conspicuous feature is an increasing trend of local fires that shows 6 peaks (Fig. 3) and then decreased at ca 870 CE. The local fires were most possibly caused by the local Slavic societies, whose economy is described as mixed, with agriculture and husbandry supplemented with hunting. In ca. 1050 CE, the area was incorporated into the Polish State; however, the human impact in this phase was still low. Local fire intensity was low until 1150 CE together with the signs of increasing local deforestation. Microcharcoal suggests an increasing trend in regional fires and through the denser settlement. It is assumed that the population of *Terra Lubucensis *in the Piast period doubled considering the tribal period but remained at a low level. The increase in the level of rural and proto-urban settlement relates to the annexation of *Terra Lubucensis *by the March of Brandenburg in the middle of the thirteenth century. This growth is mirrored in accelerating deforestation in ca 1300 CE, which is apparent primarily by the decrease in *C. betulus*. The decline of this species (and all broadleaved trees) indicates the twilight of pristine hornbeam forest and abrupt land-use change associated with growing pressures from woody biomass extraction related to local economy. For ca 300 years (1050–1349 CE), the area was managed first by the Polish State and then by Margraves of Brandenburg. The latter began intensive colonization; however, the most dramatic nature disturbances appeared in the next centuries.

It is also important to mention that the mesotrophic mire possessed shallow open water conditions that ended together with the increasing landscape openness. Thus, a strong link between the forest structure and the wetland ecosystem has never been documented in such detail. A comparable example is provided by^24^ that described increased catchment erosion and nutrient loading that are commonly recognized impacts of deforestation on wetlands. The authors suggest that deforestation increases water tables and leads to lower retention; therefore, reforestation may drastically alter this water balance, and in some cases, the protected wetland will cease to exist. However, by using our paleoecological record, it was already shown that many different scenarios are possible^51^, and deforestation does not always mean higher water table in wetlands^27^, but it may also lead to terrestrialization. In the case of this study, open water disappeared together with clearcutting that disturbed hydrology and transformed soils surrounding the mire. Despite this change, the water table was high enough to sustain the rapid peat growth in the Joannites stage; however, the ecological state of the wetland was completely different.

#### Joannites 1350–1812 CE and post-Joannites 1812–1950 CE

**Figure 4:**
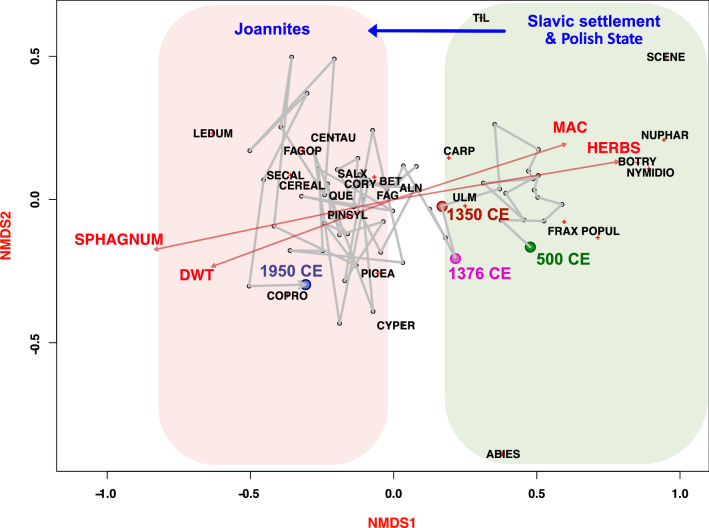
The biplot of NMDS showing the main gradients of change in Pawski Ług record. Trajectory of transformations in local vegetation starting at 500 CE, driven by humans and associated with different types of management in relation to environmental variables (DWT—depth to the water table (cm) and MAC—macroscopic charcoal influx [particles/cm^2^/year]), and general vegetation structure in the peatland (*SPHAGNUM*—peat mosses, and HERBS—herbaceous vegetation). Axis 1 represents increasing human pressure/disturbance, and the red line represents a critical transition at 1350 CE from forest vegetation to agriculture, with temporary forest regeneration at 1376 CE, which finally led to gradual forest clearance and landscape fragmentation. Axis 1 reveals a strong link between peatland hydrology, vegetation structure, and pre-Joannites local forest fires. PINSYL—*Pinus sylvestris*, BET—*Betula*, ALN—*Alnus*, SALX—*Salix*, QUE—*Quercus*, FAG—*Fagus*, CORY—*Corylus avellana*, CARP—*Carpinus betulus*, TIL—*Tilia*, PICEA—*Picea abies*, ABIES—*Abies*, ULM—*Ulmus*, FRAX—*Fraxinus excelsior*, POPUL—*Populus*, SECAL—*Secale cereale*, CEREAL—*Cereals*, FAGOP—*Fagopyrum*, CENTAU—*Centaurea cyanus*, CYPER—*Cyperaceae*, NUPHAR—*Nuphar*, NYMIDO—*Nymphaeaceae idioblasts*, LEDUM—*Ledum palustre*, BOTRY—*Botryococcus*, SCENE—*Scenedesmus*, COPRO—*Coprophilous fungi*.

The time after 1300 CE begins an irreversible critical transition in the records of Pawski Ług from the disappearing forest of the Slavic tribal past to the open landscape of the growing economy. After this date, increasing cereal abundance is visibly associated with the gradual deforestation. Moreover, since ca. 1350 CE, there were no local fires, which was most possibly caused by the lower local wood/fuel availability. Simultaneously, regional fires (represented by microcharcoal) had an anthropogenic origin and were related to regular wood usage, while peaks might represent wars and battles. Abruptly increasing openness implied ecological revolution and a tipping point for the vegetation, local people, and wetland ecosystem. A similar loss of the close-to-pristine nature in the late Middle Ages was inferred in many locations of Europe and the rest of world, and it was caused by different societies/nations^52,53^. Details of each of those studies are very complex as intricate drivers relate to historical and economic transitions. A reliable example is the impact of Polish State development^54^ and Teutonic knights’ expansion^55^ in the late Middle Ages. Here, the feudal economy and intensified settlement effectively destroyed pristine forests and less economically developed cultures (usually pagan). In general, we can state that feudal culture, with its focus on colonization, destroyed pristine nature in CE Europe. A contrasting example is the NE Poland where the human impact was low until the last and intensified ca 1600 CE^30^; however, nearly all pristine temperate forests in Europe were cut, with only some exceptions^56,57^. The process of destruction can be exemplified by the history of severe impact of the Joannites’ economy on the vegetation. Forest cutting that begun during the Polish State expansion increased after the annexation of *Terra Lubucensis *by the March of Brandenburg and intensified after the establishment of the Knights Hospitaller in nearby Łagów. All this was ultimately responsible for the cascading shift from the moderately rich to the poor part of the gradient as the result of the catchment clearance. Not only open soils and lack of the forest but also completely different microclimate and catchment hydrology affected the Pawski Ług ecosystem. The switch from mesotrophic to oligotrophic conditions indicated by *Sphagnum* invasion was triggered by rapid local deforestations. The abundance of the broadleaved tree species collapsed. Only 200 years were enough to completely eliminate the forest possessing pristine characteristics. Figure 4 reveals Trajectory of main transformations in local vegetation starting at 500 CE that are related to emergence of novel ecosystems.

Rapid deforestation was an effect of colonization under Brandenburgians and Joannites when the organization of villages and their economy were modernized. In Joannites’ estates, a model of Stadt-Landkolonisaation was implemented, in which the town of Łagów served as a commercial and craft center for the surrounding villages. The land given to the Knights Hospitaller was divided into two parts (domain bipartite): a small part remained under the direct management of the Joannites, while the majority was given to peasants for use. Large farms of at least one mansus (17–23 ha) were established, and the three-field crop rotation system, heavy plows, iron harrows, and money economy were introduced. The surviving revenue records of the Lubusz diocese from 1405 CE show the size and organization of this part of the property of the Order of St. John. The villages belonging to the Order were very large, and the peasants who inhabited them cultivated up to 64 *mansi*. Additionally, four *mansi* were usually allocated for the upkeep of local churches and parish priests^58^. The peasants paid rent to the Joannites for the land in their possession, and the Order also kept its manors in Granów and probably in Łagów. From 1600 CE, meadows and pasture forests (with oaks) with some croplands were surrounding the peatland. This kind of agriculture pattern was then sustained by the Joannites through the next 400 years.

The visual negative correlation between deforestation events and mire acidification is a remarkable feature, and it might be related to humic acid pulses from the surrounding eroded soils. Even a short event associated with *C. betulus* regeneration ca. 580–520 BP is reflected in *Sphagnum* decrease. The presence of *Sphagnum* appears to be closely associated with the catchment processes, which was probably related to flushes of humic acids from the surrounding eroded soils. This is the first record with so close deforestation-mire response pattern. Over the last 500 years, the mire was most possibly surrounded by the open fields and dispersed *Pine* forest. Increased abundance of coprophilous fungi spores and cereal pollen grains suggests croplands and grazed meadows. Stable population and settlement structures continued in the area over several hundred years until the nineteenth century, as shown on the maps of David Gily from 1802 to 1803 AD. The moments of crisis over this period, however, are visible in historical sources and in charcoal signal. The change in land-use structure is apparent in the pollen data that revealed afforestation by *Betula* and *F. sylvatica*ca. 1800 CE. After that time, the land management changed. The monastery's unified economic policy was replaced by different land management strategies by different owners who took over the monastery’s property after the dissolution of the Joannites. Consequently, in the first half of the nineteenth century, the production of local agriculture diminished. For Pawski Ług, the date 1350 CE implies the verge of the loss of pristine forests in CE Europe and entering societies from the forest tribe to the state period and feudal economy. Local deforestations cumulatively had global consequences for the soil’s transformation and wetland ecosystems that also suffered from these disturbances, which still possess the legacy effects^24^ having the origin from the late Middle Ages. It was also recently described that forest community responses to climate change are most closely related to microclimate change^59^. Past woody biomass extraction in medieval around Łagów affected the local microclimate and finally affected not only forest microclimate but also wetland functioning.

The water table in the mire decreased locally; however, we propose two alternative explanations. The water table decreased considerably in the peatland; therefore, ombrotrophic conditions became prevalent, and the wetland was colonized by *Sphagnum* or the fen was acidified^60^, which allowed *Sphagnum* to establish a floating mat that was accumulating in the peat in the stable hydrological conditions. This kind of deforestation-generated acidification was also observed in one peat core in Tuchola Pinewoods (N Poland), where it was associated with deforestation and pine planting that subsequently triggered *Sphagnum* expansion^61^. In the other case, *Sphagnum* expansion is related to the drainage and forest management (*Pinus sylvestris* plantations) in W Poland^62^. The only tree species that increased abundance around the mire, probably through the secondary succession, was *Pinus sylvestris. *Consequently, the soil experienced progressive soil acidification that also affected Pawski Ług and supported *Sphagnum* development in the basin. *Sphagnum* prefers acid conditions to grow, and it is also an important ecosystem engineer that acidifies habitat by itself^63^. However, we are sure that the mire has never been minerotrophic again and became bog until presently. The establishment of *Sphagnum* was subsequently responsible for the rapid peat accumulation that provided an exceptionally high-resolution archive of the past changes in the last 600 years. Considering stable hydrology, we would rather choose the second explanation with the floating mat development and local acidification. If it was the dry shift, we could expect more pronounced dry phases that were not existing in the profile and the peat was growing fast (2.6 mm/year). Several examples of floating mats with fast PAR were reported for Polish and European peatlands, e.g., Dury Lakes (10 mm/year)^64^, Mukrza (4.6 mm/year)^65^ as well as in Central Italy (5–6 mm/year)^66^ or Jaczno (1.38 mm/year)^30^. The stable and high ground water table in the floating *Sphagnum* mat conditions explains the rapid and constant peat growth in Pawski Ług mire.

After 1812 and Joannites’ dissolution, a secondary succession of the forest with beech occurred, and intensive agriculture in the region was abandoned. Post-Joannite estates were first taken over by the Prussian state and later by some Prussian aristocratic families. The system of large manor farms, producing for sale, was maintained, but the local economy declined due to the exclusion of Łagów from the plans for new transport routes. Industrialization processes were not established there, and depopulation due to migration to the western parts of Germany affected the region^67^. The change came at the end of the nineteenth century, when Łagów—the former seat of the Joannites—became a tourist resort. The park and game reserve, which had been created by the Joannites, were developed, and finally, in 1909, a railway connection was established.

Our study provides a high-quality reconstruction of the impact of economy on the forest and wetlands. We investigated the past transitions from the primeval forest managed by Slavic tribes to the agriculture under Brandenburgians and Joannites. The novel landscape led to the development of the novel *Sphagnum*-dominated wetland ecosystem. We suggest that the anthropogenic ecosystems are often regarded as close to pristine. Our study provides the arguments to discuss the timing of the Anthropocene period^5^ and emergence of the novel ecosystems^68^.

## Methodology

### Coring

The 5-m core was sampled in spring 2016 by using a Wardenaar sampler to recover a sample from the top 1 m. The remaining part of the profile was extracted using an INSTORF sampler (1 m long and 80 mm diameter). Because the first meter of the peat was too unconsolidated (soft), it was not possible to retrieve it using any peat corer; hence, we decided to perform the analyses only on the section between 100 and 500 cm. The samples were transported to the laboratory and stored frozen. The material was subsampled from the de-frosted core for each proxy and dated and analyzed at a range of resolutions. Pollen, microscopic charcoal, and TA were sampled every 5 cm, whereas plant macrofossils and macroscopic charcoal were analyzed contiguously every 1 cm.

### Radiocarbon dating and chronology

The absolute chronology was based on 39^14^C AMS dates provided by the Poznań Radiocarbon Laboratory (Poland) (Table 1). The age-depth model was calculated using the OxCal 4.3 software^69–71^}. The IntCal13 atmospheric curve was used as the calibration dataset^72^.

**Table 1:** The list of radiocarbon dates from Pawski Ług peatland with calibration.

N	Lab ID – sample number	Depth [cm]	^14^C date [^14^C yrs BP]	Error [^14^C yrs]	Calibrated ages [cal. BP] (2σ 95.4%)	Material dated
1	Poz-95865	100,5	100.35 pMC	0,32pMC	1947–1958	*Sphagnum* stems
2	Poz95866	109,5	70	30	1875–1926	*Sphagnum* stems
3	Poz-95867	119,5	150	30	1815–1888	*Sphagnum* stems
4	Poz-95868	129,5	200	30	1775–1825 (84.5%) 1830–1858 (10.9%)	*Sphagnum* stems
5	Poz-95869	139,5	180	30	1794–1808	*Sphagnum* stems
6	Poz-95870	149,5	155	30	1725–1779	*Sphagnum* stems
7	Poz-95871	159,5	100	30	1700–1758	*Sphagnum* stems
8	Poz-96004	169,5	150	30	1681–1734	*Sphagnum* stems
9	Poz-96006	179,5	160	30	1666–1703	*Sphagnum* stems
10	Poz-96007	189,5	205	30	1653–1682	*Sphagnum* stems
11	Poz-95957	200,5	245	30	1634–1665	*Sphagnum* stems
12	Poz-95958	209,5	285	30	1612–1652	*Sphagnum* stems
13	Poz-96010	219,5	290	30	1577–1636	*Sphagnum* stems
14	Poz-96011	229,5	300	30	1550–1612	*Sphagnum* stems
15	Poz-96012	239,5	250	30	1532–1586	*Sphagnum* stems
16	Poz-96013	249,5	295	30	1515–1566	*Sphagnum* stems
17	Poz-96014	259,5	330	30	1494–1545	*Sphagnum* stems
18	Poz-96015	269,5	300	30	1475–1524	*Sphagnum* stems
19	Poz-96016	279,5	370	30	1452–1495	*Sphagnum* stems
20	Poz-96017	289,5	430	30	1431–1468	*Sphagnum* stems
21	Poz-96020	299,5	470	30	1406–1441	*Sphagnum* stems
22	Poz-96021	309,5	650	30	1351–1393	*Sphagnum* stems
23	Poz-96022	319,5	550	30	1305–1350	*Sphagnum* stems
24	Poz-96023	329,5	765	30	1215–1280	*Sphagnum* stems
25	Poz-96024	339,5	980	30	1079–1157	*Sphagnum* stems
26	Poz-96085	349,5	995	30	983–1054	*Sphagnum* stems
27	Poz-96087	359,5	1105	30	871–983	*Sphagnum* stems
28	Poz-96088	369,5	1300	30	669– 761	*Sphagnum* stems
29	Poz-96089	379,5	1465	30	549–641	*Sphagnum* stems

Historical data on the surrounding area was extracted from the available medieval and early modern written sources (chronicles, inventories, old maps) and historical and archeological studies, which were analyzed by traditional historical, spatial, and statistical methods.

### Palynological analysis

A total of 80 samples (2 cm^3^ in volume, sampled every 5 cm) were prepared using standard laboratory procedures for palynological analysis following standard procedures^73^ and using *Lycopodium* marker. Pollen, cryptogam spores, and selected nonpollen palynomorphs (NPPs) were counted under a binocular microscope until the total pollen sum (TPS) in each sample reached at least 500. Pollen grains were identified using atlases and keys^74–76^. The results of the palynological analysis were expressed as percentages calculated on the basis of the ratio of an individual taxon to the TPS, i.e., the sum of arboreal pollen (AP) and nonarboreal pollen (NAP) excluding aquatic and wetland plants including Cyperaceae, and cryptogams. For better understanding, some pollen taxa that are human impact indicators were grouped into cultivated land indicators and major ruderals^77–79^.

### Charcoal analysis

Microscopic charcoal particles (size: > 10 μm) were counted from the same slides as pollen and NPPs^80^ until the number of charcoal particles and *Lycopodium* spores, counted together, exceeded 200^81^. The calculations of microscopic charcoal accumulation rates (MIC) follow the formula proposed by^82^, i.e., MIC = Ct × PAR, where Ct is the concentration of charcoal particles (unit: particles/cm^2^/yr)^80^.

For macroscopic charcoal analysis, 400 contiguous samples (1 cm^3^) were prepared by bleaching to create a more visible contrast between the charcoal and the remaining organic matter and by wet sieving through a 100-μm mesh following the method described by^83^. The particles were divided into 4 fractions, and the entire sample was analyzed under 60× magnification. Macroscopic charcoal influx (proxy for local fires^84^) or accumulation rates (MAC, particles/cm^2^/year) were calculated using the charcoal concentrations and PAR.

### Plant macrofossils analysis

The macrofossil composition of the 400 contiguous peat samples (volume: ≈3 cm^3^) was determined by sieving each sample through a 125-μm diameter mesh. Plant remains were later scanned using a binocular microscope (10 × –50 × magnifications) and identified using an extensive reference collection of type material^85^. Volume percentages were estimated for all components except for seeds, *Betula* spp. catkin scales, *Eriophorum vaginatum* spindles, *Carex* spp. nutlets, *Chara* oospores, and *Sphagnum* spore capsules, which were counted and expressed as the number (n) present in each subsample.

### Testate amoebae analysis

Peat for TA analysis (2 cm^3^ in volume) was sampled from the same depths as those for pollen and microscopic charcoal analyses. Peat samples were washed under 0.3-mm sieves following the method described by^86^. TA were analyzed under a light microscope between 200 × and 400 × magnification, with a minimum of 100 tests per sample^87^. Several keys and taxonomic monographs^88–93^ as well as internet resources^94^ were used to achieve the highest possible taxonomic resolution. The results of the TA analysis were used for the quantitative DWT reconstructions^46^.

### Statistics and diagrams

Diagrams with paleoecological proxy data were plotted using C2^95^ or Tilia graph^96^ software. To adjust the description of the proxy data, we divided each diagram into zones based on the pollen and NPP spectra, as these proxies represent the broadest spatial set of environmental changes. To draw the synthesis figure, DataGraph was used^97^. The final graphical output was edited using Affinity Designer (affinity.serif.com). Quantitative reconstruction of the TA-based DWT was performed in C2 software^95^ by using a training set developed for northern Poland by^46^. The local training set was used to avoid biogeographical and taxonomic bias^98,99^. Nonmetric Multidimensional Scaling (NMDS) on the Bray–Curtis dissimilarity was applied using the *vegan* package^100^ to explore trajectory of changes over time in vegetation in relation to environment. Selected environmental variables (DWT, MIC, MAC, vascular plants, and Sphagnum) were fitted onto the ordination post hoc with the *envfit* function. Numerical analyses were performed using R version 3.6.2^101^.

## Supplementary information

Supplementary Legends.

Supplementary Information 2.

Supplementary Information 3.

Supplementary Information 4.
