# Supplementary material for: How Joannites’ economy eradicated primeval forest and created anthroecosystems in medieval Central Europe
Source: Sci Rep. 2020 Nov 19;10:18775. doi: 10.1038/s41598-020-75692-4 (PMC7677315; doi:10.1038/s41598-020-75692-4)
Supplement: Supplementary file 1 — Supplementary Legends. [file 41598_2020_75692_MOESM1_ESM.docx]

**Supplementary figures**

Supplementary Figure S1. Pollen percentage diagram – drawn with Tilia software (https://www.tiliait.com). Figure constructed by ML with Affinity Designer (https://affinity.serif.com).

Supplementary Figure S2. Plant macrofossil diagram – drawn in C2 software (https://www.staff.ncl.ac.uk/stephen.juggins/software/C2Home.htm). Figure constructed by ML with Affinity Designer (https://affinity.serif.com).

Supplementary Figure S3. Testate amoebae percent diagram with quantitative estimates of the depth to the water table – drawn in C2 software (https://www.staff.ncl.ac.uk/stephen.juggins/software/C2Home.htm). Figure constructed by ML with Affinity Designer (https://affinity.serif.com).
