# Supplementary figures and images for: How Joannites’ economy eradicated primeval forest and created anthroecosystems in medieval Central Europe
Source: Sci Rep. 2020 Nov 19;10:18775. doi: 10.1038/s41598-020-75692-4 (PMC7677315; doi:10.1038/s41598-020-75692-4)

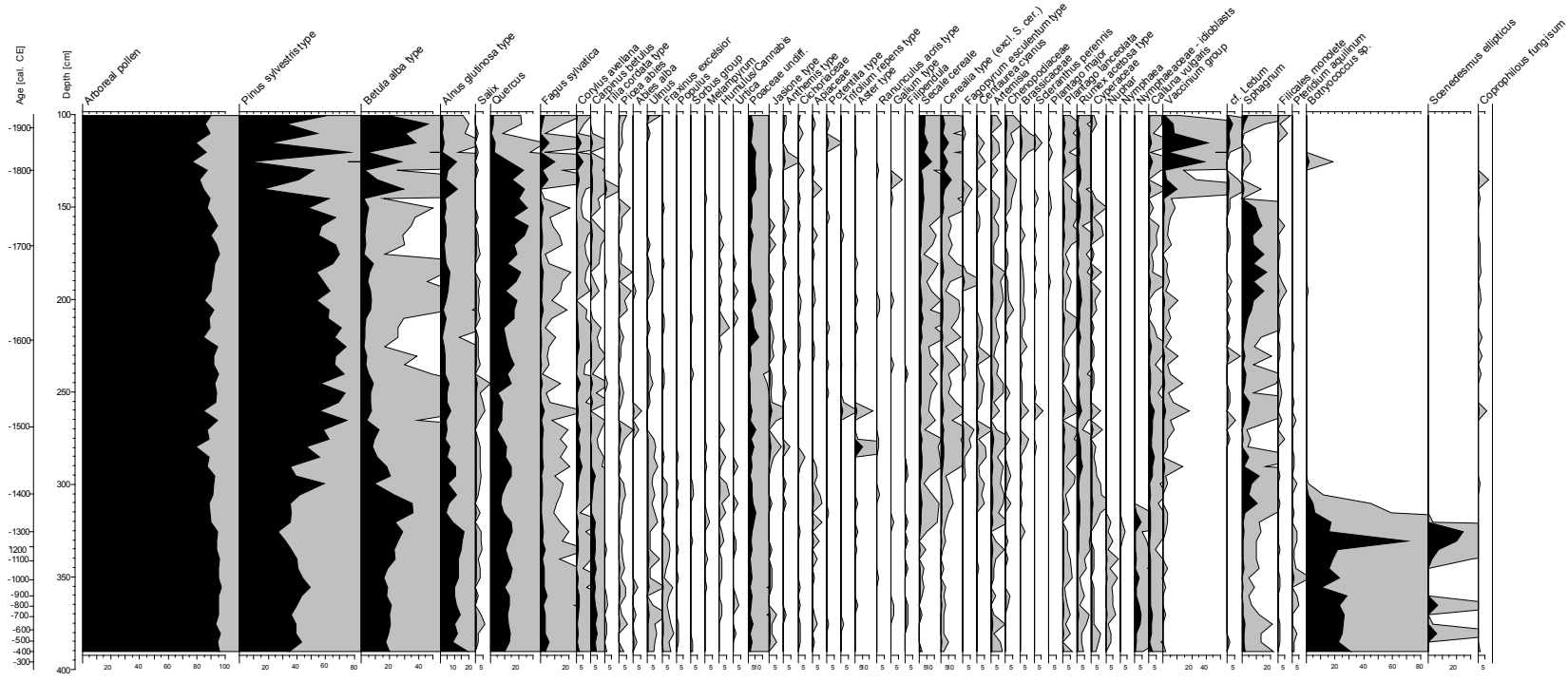

Supplement: Supplementary file 2 — Supplementary Information 2. [file 41598_2020_75692_MOESM2_ESM.pdf]

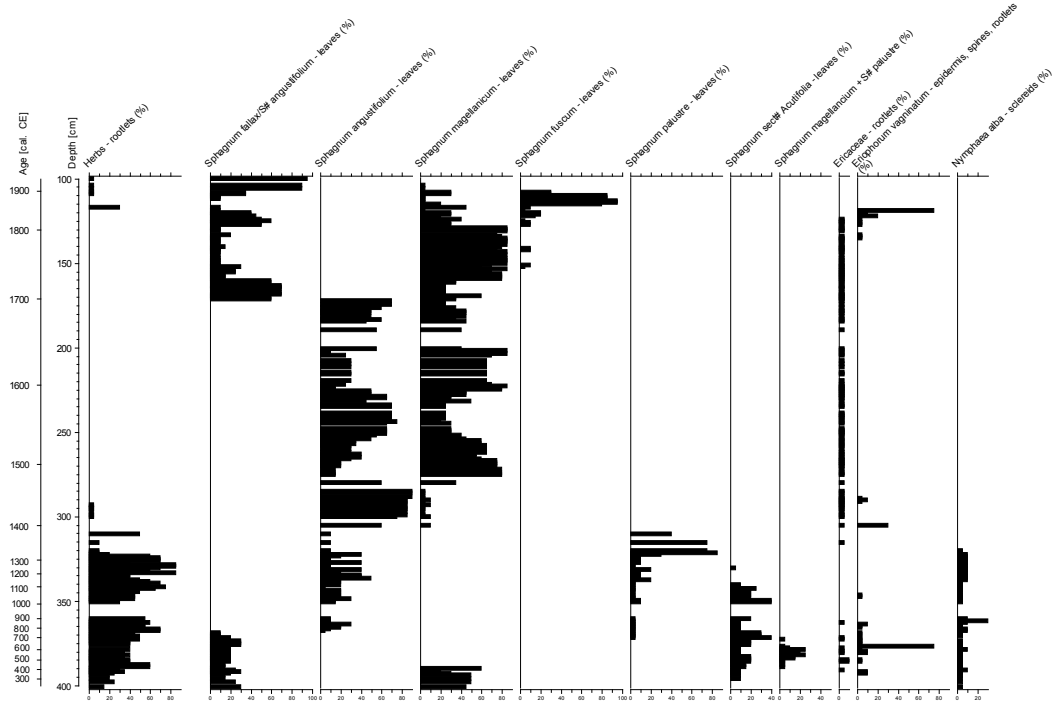

Supplement: Supplementary file 3 — Supplementary Information 3. [file 41598_2020_75692_MOESM3_ESM.pdf]

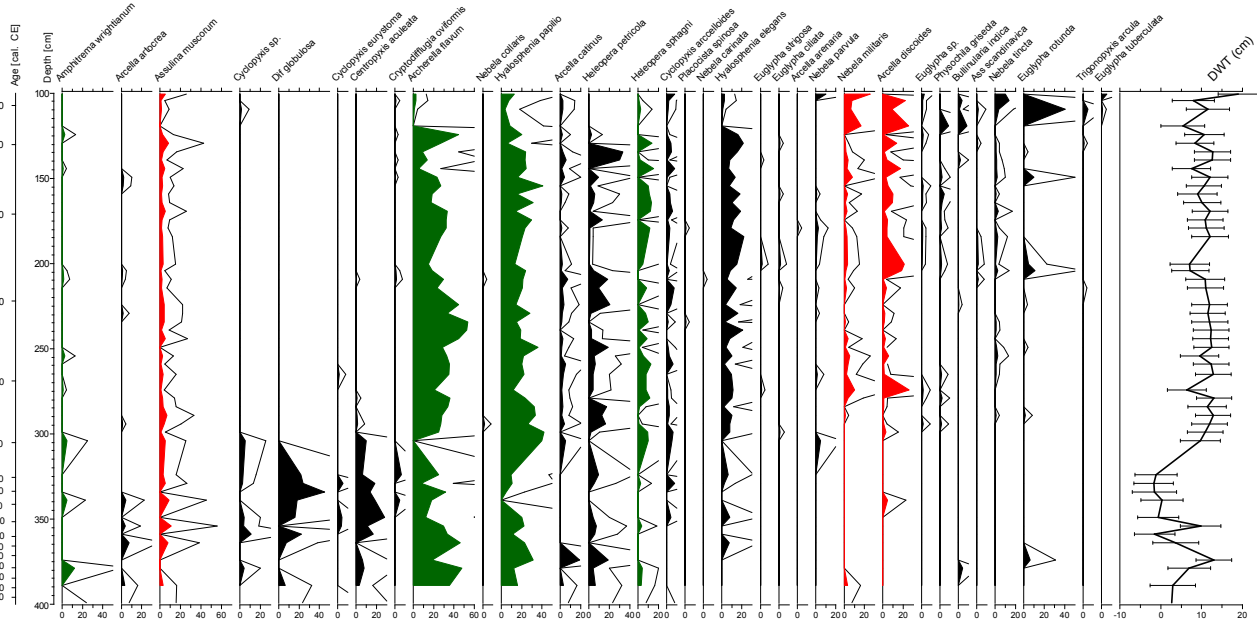

Supplement: Supplementary file 4 — Supplementary Information 4. [file 41598_2020_75692_MOESM4_ESM.pdf]
